# To become more sustainable organic agriculture needs genome editing technology

**DOI:** 10.3389/fbioe.2022.912793

**Published:** 2022-08-19

**Authors:** Patricia Machado Bueno Fernandes, Luíza Favaratto, A. Alberto R. Fernandes, Carmen Vicien, Deise M F Capalbo, Francisco Murilo Zerbini

**Affiliations:** ^1^ Biotechnology Core, Federal University of Espírito Santo, Vitória, Brazil; ^2^ Faculty of Agronomy, University of Buenos Aires and Institute for Scientific Cooperation in Environment and Health, Buenos Aires, Argentina; ^3^ R&D Department, Embrapa Environment, Jaguariúna, Brazil; ^4^ Department of Plant Pathology, Federal University of Viçosa, Viçosa, Brazil

**Keywords:** global warming1, CRISPR2, food insecurity3, food regulation4, market release5

## 1 Introduction

Worldwide, the area identified as “organic agriculture” comprises ca. 72.3 million hectares, with an average yearly growth of 10%. In 2019 the global market of organic foods and drinks reached more than 106 billion euros (FAO 2021). With this area and growth, organic agriculture is already an important player in global food production. Nevertheless, the positive environmental effects of organic farming are less evident when considering food production in kilograms rather than per hectare of cultivated land, mostly because of lower crop yields due to several factors. This leads to the necessity of more land in the case of organic farming, compared to the traditional way, to obtain a similar amount of food as an output (Willer et al., 2021).

In general, regulations of organic production exist under the umbrella of a larger framework of public policies aimed at the adoption of sustainable agricultural practices and the conservation of agroecosystems, focused on food and nutritional security of the population, fairer trade relations, and conscious consumption. Agriculture is heavily affected by the climate crisis, while also representing one of the major sources of greenhouse gas emissions (UNF 2021). The internationally recognized greenhouse gasses covered under the United Nations Framework Convention on Climate Change include carbon dioxide (CO_2_), methane (CH_4_), nitrous oxide (N_2_O), and carbon monoxide (CO). The Gas Emission Estimation System (SEEG 2022) shows that agriculture has a prominent role in the emissions of those greenhouse gasses, especially CH_4_ and N_2_O.

The world population is predicted to reach 9.7 billion in 2050 (United Nations 2019). According to the World Hunger Clock, in March 2022, approximately 2.4 billion people live in moderate and severe food insecurity. That food production must increase in order to fight this foreseen insecurity is self-evident, but this needs to be done while also ensuring the achievement of the Sustainable Development Goals (SDG). Incorporating new technologies is one major way of reaching this objective and helping to solve the climate crisis.

## 2 Relationship between organic agriculture and biotechnology

Historically, the relationship between organic agriculture and biotechnology has been antagonistic (Husaini and Sohail 2018). Indeed, a true ideological war has been pursued for years between supporters of organic versus biotechnological agriculture. This antagonism induced many smallholder farmers to believe that there is a complete incompatibility between the two agricultural systems (Purnhagen et al., 2021). This struggle resulted in a legal framework for organic farming which prevents farmers from incorporating GMOs into their production systems, even if it would allow for better quality, increased climate-related resilience, and productivity, and even less use of pesticides. As a result, organic farmers view biotechnology as unnatural and opposed to the principles that drive organic agriculture (IFOAM 2016).

Biotechnology is thus associated with industrial, commodity-based farming, monoculture, intensive use of pesticides, and patented seeds. One of the biggest misconceptions of the organic foundation is to confuse biotechnology - a production process - with an intrinsically unsafe and hazardous product. This misconception is in large part the result of the extreme regulatory framework to which biotech crops are subjected in most countries. In Brazil, for example, obtaining a permit for the “planned release” of most GM plants requires (among other things) detailed information on the dissemination of GM pollen into the environment, on all plant species with which the GM species could possibly cross, and the long-term effects of such crosses. Requirements for a commercial release are orders of magnitude more complex. This difficulty seems to be a constant in most countries. In the European Community, China, and Japan, important players in this subject, there are even more restrictive requirements. It is essentially impossible for an overworked researcher in an understaffed public university or research institute to satisfy all these requirements. Thus, only the large agribusiness companies, with fully staffed compliance departments and plentiful resources, are capable of obtaining such permits. The unfortunate outcome of this ideological war is an aversion and prohibition of GM crops which in reality could be extremely helpful and are completely compatible with organic, sustainable agriculture, and which have no detectable differences regarding food or environmental safety.

## 3 CRISPRized plants to organic farming

**FIGURE 1:**
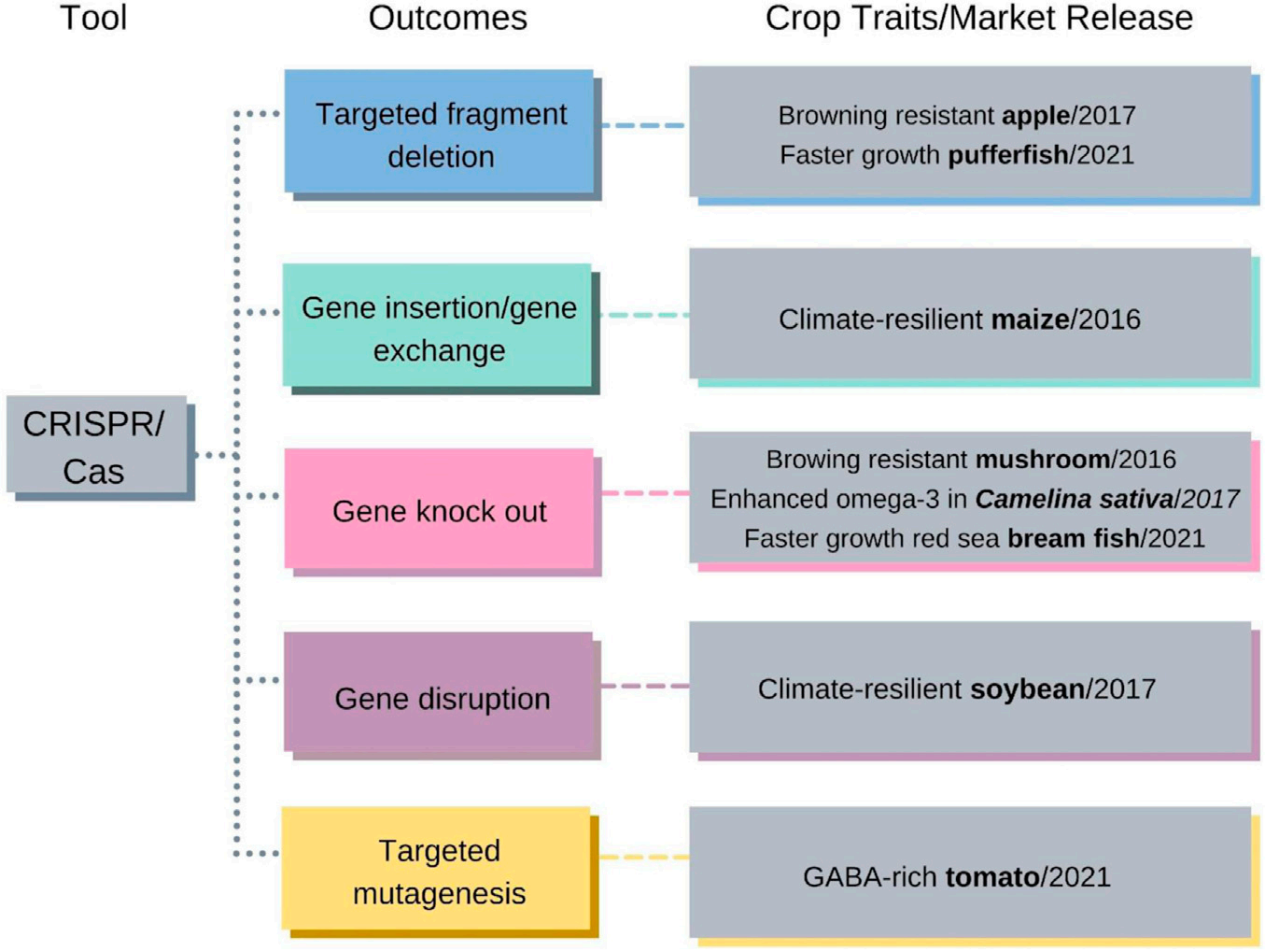
CRISPR/Cas and its outcomes and characteristics of crops already available on the market. Colored arrows and boxes link published crop trait examples and year of market release with the associated genome-editing tool and outcome (Jansing et al., 2019). Copyright 2019, MDPI, Basel, Switzerland.

A new window of opportunities for organic agriculture presents itself with the advent of gene-editing technologies such as CRISPR-Cas9. Clustered, regularly interspaced short palindromic repeats (CRISPR) - associated proteins is a technology for genome editing that enables the knock-in and/or knock-out of target genes in specific genome regions (Doudna and Charpentier 2014). This strategy has been successfully applied in model plants, such as Arabidopsis and tobacco, and in crops, as presented in Figure 1, to modify endogenous protein-coding genes (GLP 2020).

It is known that mutagenesis may occur naturally or through long processes of genetic selection. The CRISPR-Cas9 technique made gene editing possible with the purpose of inducing important properties in plant development without necessarily introducing an exogenous gene (Waltz 2016a, 2016b; Nishitani et al., 2016; Shi et al., 2017; Waltz 2018; Jansing et al., 2019; Lyzenga et al., 2019; Gramazio et al., 2020; Cai et al., 2022). In this way, this biotechnological tool eliminates one of the major points raised against biotech crops, which is the “unnatural” insertion of an exogenous gene into the plant’s genome. It is imperative to note that, to date, no commercial platform exists enabling the detection of CRISPR-Cas-induced genome edits. Thus, genome editing through CRISPR-Cas is a way of accelerating the production of improved cultivars in a completely safe and sustainable fashion. As national and supranational regulators (such as the Brazilian CTNBio and CONABIA in Argentina, and the European Commission, respectively) engage in debates on whether (and how) to regulate crops obtained with the use of CRISPR-Cas-based and other genome-editing technologies, it is imperative that the nature of genome editing be understood, to avoid the same mistakes made when regulating GM crops, of introducing excessive (and unnecessary) regulations which prevent the widespread use of the technology beyond a few major commodities. To deny the benefits of this revolutionary technology to organic and smallholder farmers would be a tragedy of immense proportions.

## 4 The way forward–can biotechnology and organic agriculture become partners instead of enemies?

Forty years after the first GM product came on the market (human insulin produced in bacteria; Itakura et al., 1977), the discussion about the safety of GMOs still reverberates. In the 1980’s, the first transgenic tobacco, maize, and wheat plants appeared in the United States, and in 1994, the first GM food (the Flavr Savr™ tomato) arrived in American supermarkets (Kramer and Redenbaugh 1994). 30 years later, despite growing scientific evidence that GMOs are as safe as conventional crops–and in fact can bring important benefits for food security and the environment–they remain rejected by organic regulations. This situation represents a true predicament for the advancement of organic farming (Husaini and Sohail 2018).

To cite one of the many statements around the safety of products from modern biotechnology and their potential to help in SDG and overcome environmental problems, a recent study in Spain (Vega Rodríguez et al., 2022) showed that GMOs can serve as nutraceuticals and edible vaccines without the need for broad-scale industrial facilities for production. Thus, genetically edited foods need to be treated as traditional foods, and food security needs to be prioritized over the methods by which genetic modification/edition traits and properties were incorporated. The researchers also emphasized that debates over modern foods should be based on scientific evidence rather than emotions. Consumer health benefits need to be made known to the public to dispel skepticism related to biotechnology.

There is an urgent need to provide mechanisms so that scientific and technological knowledge is available to all, including the organic farmers and consumers who could benefit significantly from the application of the newest genome-editing technologies to crop improvement. If biotechnology and organic agriculture become partners, both will benefit. But the ultimate winner will be the general population, who will have access to food products that are nutritional, safe, and produced in a sustainable fashion.

CRISPR technology provides the perfect opportunity for this partnership to happen. It is easy to implement, affordable, and, if regulatory hurdles are not unfeasible, its derived seeds will be viable for small family farmers, the basis of organic agriculture. The CRISPR genome editing technology is not only equivalent to traditional breeding technique but actually much more controlled and faster. It should be embraced by the adepts of organic agriculture. We believe that the long-overdue partnership between biotechnology and organic agriculture is fundamental for the mitigation of food insecurity and is the only way forward to a truly sustainable agriculture (World Hunger Clock, 2021).
